# Amino Acid Complexes of Zirconium in a Carbon Composite for the Efficient Removal of Fluoride Ions from Water

**DOI:** 10.3390/ijerph19063640

**Published:** 2022-03-18

**Authors:** Efrén González-Aguiñaga, José Antonio Pérez-Tavares, Rita Patakfalvi, Tamás Szabó, Erzsébet Illés, Héctor Pérez Ladrón de Guevara, Pablo Eduardo Cardoso-Avila, Jesús Castañeda-Contreras, Quetzalcoatl Enrique Saavedra Arroyo

**Affiliations:** 1 Centro Universitario de los Lagos, Universidad de Guadalajara, Lagos de Moreno 47460, Jalisco, Mexico; efren.gonzalez5990@alumnos.udg.mx (E.G.-A.); ptavaresjose@gmail.com (J.A.P.-T.); hperez@culagos.udg.mx (H.P.L.d.G.); jesus.castaneda@academicos.udg.mx (J.C.-C.); 2 Department of Physical Chemistry and Materials Science, University of Szeged, Rerrich Béla tér 1, 6720 Szeged, Hungary; 3 Department of Food Engineering, University of Szeged, 6720 Szeged, Hungary; illese@chem.u-szeged.hu; 4 Centro de Investigaciones en Óptica A.C., León 37150, Guanajuato, Mexico; pecardoso@cio.mx; 5 Instituto Tecnológico Superior de Irapuato, Irapuato 36821, Guanajuato, Mexico; enrique.sa@irapuato.tecnm.mx

**Keywords:** fluoride adsorption, zirconium complex, amino acid, graphite oxide

## Abstract

Amino acid complexes of zirconia represent an entirely new class of materials that were synthesized and studied for the first time for the decontamination of fluoride ion containing aqueous solutions. Glutamic and aspartic acid complexes of zirconia assembled with thin carbon (stacked graphene oxide) platelets deriving from graphite oxide (GO) were synthesized by a two-step method to prepare adsorbents. The characterization of the complexes was carried out using infrared spectroscopy to determine the functional groups and the types of interaction between the composites and fluoride ions. To reveal the mechanisms and extent of adsorption, two types of batch adsorption measurements were performed: (i) varying equilibrium fluoride ion concentrations to construct adsorption isotherms at pH = 7 in the absence of added electrolytes and (ii) using fixed initial fluoride ion concentrations (10 mg/L) with a variation of either the pH or the concentration of a series of salts that potentially interfere with adsorption. The experimental adsorption isotherms were fitted by three different theoretical isotherm equations, and they are described most appropriately by the two-site Langmuir model for both adsorbents. The adsorption capacities of Zr-glutamic acid-graphite oxide and Zr-aspartic acid-graphite oxide are 105.3 and 101.0 mg/g, respectively. We found that two distinct binding modes are combined in the Zr-amino acid complexes: at low solution concentrations, F^−^ ions are preferentially adsorbed by coordinating to the surface Zr species up to a capacity of ca. 10 mg/g. At higher concentrations, however, large amounts of fluoride ions may undergo anion exchange processes and physisorption may occur on the positively charged ammonium moieties of the interfacially bound amino acid molecules. The high adsorption capacity and affinity of the studied dicarboxylate-type amino acids demonstrate that amino acid complexes of zirconia are highly variable materials for the safe and efficient capture of strong Lewis base-type ions such as fluoride.

## 1. Introduction

According to the World Health Organization (WHO), about 80% of diseases worldwide are related to low-quality water consumption [1]. The composition of the water depends on the geological conditions from which it is extracted. The concentration of minerals present in freshwater can vary from tens to hundreds of mg/L [2]. The fluoride ion content is associated with different minerals, such as fluorite, biotite, topaz, and granite [3,4]. The content of this fluorine species is generally found in concentrations lower than 0.5 mg/L. Generally, they have low concentrations, but they can reach extreme concentration levels in some regions of the world [2].

Many Mexican states are affected by high fluoride ion concentrations in drinking water, one of them being Jalisco. Los Altos de Jalisco (Jalisco State Heights) is characterized by groundwater with concentrations above the permissible limit (1.5 mg/L) established by the WHO and the Official Mexican Standard [2,5]. The intake of these pollutants can lead to various health conditions, such as dental fluorosis, skeletal fluorosis, neurotoxicity, allergies, and increased risk of bone fractures [2,3].

There are different fluoride ion removal techniques, which focus on chemical precipitation methods, membrane separation methodologies such as reverse osmosis or nanofiltration, ion exchange, adsorption, or electrochemical methods [6,7,8,9]. The removal of fluoride ions by adsorption has excellent potential due to its cost effectiveness, simplicity, high efficiency, and possible reuse of the adsorbent (regeneration). A wide variety of materials and their modifications have been tested for the defluoridation of water, such as activated carbon [10,11], activated alumina [12,13], iron-based materials [14,15], low-cost natural materials [16], and biomaterials [17,18].

Metal oxides such as titania, alumina, and zirconia have been shown to be effective materials for the selective removal of anions from aqueous solutions. Among these adsorbents, zirconium oxide (ZrO_2_) is one of the most widely studied materials for water defluoridation due to its high binding affinity with fluoride through the Lewis acid–base interaction [19,20]. The zirconium coordination complexes and fluoride show a strong interaction [21,22], so this could be an attractive candidate for purification of groundwater, industrial waste, and drinking water. Several zirconium-based materials have been reported, for example hybrid zirconium (IV)-hexamethylenediamine and amorphous zirconium phosphate [23], zirconium (IV)-doped polypyrrole/zirconium (IV) iodate [24], β-cyclodextrin modified hydrous zirconium oxide [25], iron (III)-zirconium(IV) hybrid oxide [26], zirconium-coated pumice [27], Zr/Fe/Al-modified chitosan beads [28], zirconium modified activated carbon fibers [29], complexes of zirconium and graphene oxide-zirconium with dicarboxylic acids [30,31], among others. Recently, low-cost carbon-based adsorbents were synthesized [32]. The Zr (IV)-impregnated graphene oxide-coated sand showed a considerable fluoride adsorption capacity compared with other carbon-based economic adsorbents. Graphene oxide, as a novel carbon material incorporated into composites with zirconium compounds, has been demonstrated to be beneficial for the detoxification of chemical warfare agents by solid/gas adsorption processes [33,34].

Zr-based coordination polymers also have applications in the field of environmental chemistry. Zr-based porous coordination polymer nanoparticles were prepared with amine-based ligand, obtaining a green, sustainable, heterogeneous, microporous catalyst for the synthesis of benzimidazole [35]. A Zr-based coordination polymer within an aqueous solution was synthesized with terephthalic acid. The mesoporous material had an excellent adsorption capacity of methylene blue [36]. Zr-based metal–organic frameworks were used as adsorbents for the efficient removal of organophosphorus pesticides from aqueous solution [37]. Zr–organic frameworks were structurally modified with chitosan, obtaining novel biocomposite spheres for effective retention of fluoride in aqueous media [38].

The use of chelating agents such as dicarboxylic acids has a positive effect on the reduction in particle size during the nucleation and aggregation stage of ZrO_2_ [30,31,39]. Activated carbon (F400) was modified with Zr (IV) together with oxalic acid and used to maximize the zirconium dispersion and enhance fluoride adsorption [39]. The maximum defluoridation capacity was 17.70 mg/g, a higher value than that of pure F400 or the simple Zr-doped activated carbon without oxalic acid. It was proposed that the fluoride adsorption mechanism included the exchange of hydroxyl groups from the Zr-oxalate surface sites and fluoride interaction with the positive charge of zirconium ions in Zr = O groups.

Another notable example of the use of dicarboxylate-functionalized adsorbents includes an oxalic acid–graphene oxide–zirconium needle-like complex, which was synthesized and used to remove fluoride from simulated fluoride-contaminated water [31]. The maximum fluoride adsorption capacity was 9.70 mg/g at the minimum contact time of 18 min. The adsorption mechanism depended on pH, with electrostatic interactions under low pH and ion-exchange mechanisms under neutral pH. However, apart from these three publications reporting on oxalic acid-modified sorbents, the literature lacks any reports that explore the use of chelating agents of various kinds for the removal of inorganic fluoride anions from aqueous solutions.

Here, we present an entirely novel material for capturing fluoride ions from solutions: amino acid complexes of zirconium on a graphite oxide support. We propose that the use of amino acids containing two carboxylate groups (aspartic and glutamic acid) is superior to the use of oxalic acid as a complexing agent, owing to the presence of the ammonium groups. The latter are envisioned to bear a significant contribution to the adsorbed amount by allowing the ion-exchange adsorption of fluoride anions.

## 2. Materials and Methods

### 2.1. Synthesis of Graphite Oxide

Graphite oxide (GO) was prepared using the improved Hummers–Offeman method [40], for which a 9:1 mixture of concentrated H_2_SO_4_:H_3_PO_4_ (360 mL:40 mL; H_2_SO_4_: ≥95–98%, Meyer, Química Suastes, S.A. de C.V., Mexico City, Mexico. H_3_PO_4_, ≥85%, Karal, León, Guanajuato, Mexico) was added to 3 g graphite flakes (<20 μm, Sigma-Aldrich Chemical Co., St. Louis, MO, USA) and 18 g KMnO_4_ (J. T. Baker, S.A. de C.V., Xalostoc, Estado de México, Mexico). The reaction was heated to 50 °C and magnetically stirred for 12 h, and then allowed to cool to room temperature. Subsequently, in a beaker, 400 mL of ice was mixed with 3 mL 30% H_2_O_2_ (Karal, León, Mexico) and added to the previous reaction mixture. When this reached room temperature, the material was washed three times by centrifugation at 6000 rpm for 2 h (Z 206 A Centrifuge, Hermle LaborTechnik GmbH, Wehingen, Germany) with distilled water, 20% HCl (≥36.5–38% Meyer, Química Suastes, S.A. de C.V., Mexico City, Mexico), and ethanol (CTR Scientific, Monterrey, Nuevo León, Mexico). The graphite oxide was dialyzed through a collagen membrane with several water changes, until conductivity of between 10 and 20 µS/cm was reached (Orion Star A215 pH/Conductivity Meter, Thermo Scientific, Waltham, MA, USA). The purified graphite oxide was stored in suspension for later use.

### 2.2. Synthesis and Characterization of Zr-Amino Acid-Graphite Oxide Compounds

Zr-amino acid-graphite oxide compounds were prepared using the chemical precipitation method [31]. The graphite oxide suspension (0.976 g, 30% *w*/*w*) was sonicated, and (100 mL, 0.1 M) zirconium oxychloride solution (Sigma-Aldrich Chemical Co., St. Louis, MO, USA) was added drop by drop with constant stirring at a temperature of 40 °C. Then, the corresponding amino acid solution (100 mL, 0.2 M), aspartic acid (Asp) or glutamic acid (Glu) (Sigma-Aldrich Chemical Co., St. Louis, MO, USA), was added in the same way. The compounds were left to age for 24 h and then centrifuged with water to eliminate any excess salts present. Finally, the compounds were dried at a temperature of 40 °C. The obtained compounds were named ZGluGO (Zr-glutamic acid-graphite oxide) and ZAspGO (Zr-aspartic acid-graphite oxide) or ZAAGO when both amino acid-containing sorbents were considered.

Infrared spectroscopic measurements were undertaken in a Fourier Transform Infrared/Far Infrared (FT-IR/FIR) spectrometer (Frontier model, Perkin Elmer, Waltham, MA, USA) using the Total Attenuated Reflection technique in the region of 4000 to 400 cm^−1^ (FT-IR) and 650 to 150 cm^−1^ (FIR) using a spectroscopic-grade polyethylene pellet (Sigma-Aldrich Chemical Co., St. Louis, MO, USA).

Thermogravimetric analysis (TGA) was carried out on Q600 apparatus (TA instruments, New Castle, DE, USA) from 25 to 1000 °C using a 10 °C/min ramp under an extra-dry air atmosphere.

Scanning electron microscopy (SEM) was performed using a JSM-7800F JEOL (Tokyo, Japan) microscope: a drop of diluted powder samples was placed on top of silicon substrate and allowed to dry at room temperature. The images were obtained using a 5 kV accelerating voltage. Energy-dispersive spectroscopy (EDS) data were acquired by an X-Max 80 spectrometer from Oxford Instruments incorporated in the SEM system.

X-ray diffraction patterns were obtained from 4 to 80 degrees using a D2 Phaser X-ray diffractometer equipped with a Cu Kα (λ = 0.154 nm) radiation source (Bruker Corporation, Billerica, MA, USA).

### 2.3. Adsorption Experiments

We studied variations in several parameters, modifying the concentration of the fluoride ion, pH, and the effect of interfering ions. The same amount of solution and mass of adsorbent material was used in all cases: 50 mL of sodium fluoride (≥99%, Jalmek Científica, San Nicolas de los Garza, Nuevo León, Mexico) solution was used and stirred with 50 mg of the adsorbent material.

The isotherm study was undertaken using centrifuge tubes with 50.0 mL of a solution containing fluoride ions with initial concentrations of 5 to 300 mg/L. The adsorbent loading was set at 1.0 g/L at a pH of ≈ 6.5–7.0. These tubes were agitated at 40 rpm (IKA Loopster digital, IKA Works, Inc., Wilmington, NC, USA) at 25 °C for 180 min. Subsequently, the suspension was filtered through a membrane of 20 µm pore diameter (Corning^®^, Corning, NY, USA) and the fluoride ion concentration was measured by an ion selective electrode (HI98402 Fluoride Meter, Hanna Instruments, Padua, Italia), mixing 25 mL of filtrate with 25 mL of Total Ionic Strength Adjustment Buffer (TISAB II). The preparation of TISAB II was as follows: 4.0 g of trans-1,2-Diaminocyclohexane-*N,N,N′,N′*-tetraacetic acid monohydrate (≥99%, Sigma-Aldrich Chemical Co., St. Louis, MO, USA), 57.0 mL of glacial acetic acid (Sigma-Aldrich Chemical Co., St. Louis, MO, USA), and 58.0 g of sodium chloride (99%, Jalmek Científica, San Nicolas de los Garza, Nuevo León, Mexico) were dissolved in 500 mL of distilled water. The pH of the dissolution was adjusted between 5.0 and 5.5 by dropwise adding of 5.0 M of sodium hydroxide. Finally, the volume of the dissolution was adjusted to 1 L by adding distilled water.

The equilibrium data obtained for the initial variation in fluoride concentration were adjusted for three models of adsorption isotherms: Langmuir, Freundlich, and two-site Langmuir isotherms with their nonlinear and linear form [41,42].

Nonlinear forms:

(1) $$\mathrm{Langmuir}\;\mathrm{isotherm}:\;q_{e}=\frac{Q_{max}^{0}K_{L}C_{e}}{1+K_{L}C_{e}}$$

(2) $$\mathrm{Freundlich}\;\mathrm{isotherm}:\;q_{e}=K_{F}C_{e}^{n}$$

(3) $$\mathrm{Two}-\mathrm{site}\;\mathrm{Langmuir}\;\mathrm{isotherm}:\;q_{e}=\frac{q_{1}b_{1}C_{e}}{1+b_{1}C_{e}}+\frac{q_{2}b_{2}C_{e}}{1+b_{2}C_{e}}$$

Linear forms:

(4) $$\mathrm{Langmuir}\;\mathrm{isotherm}:\;\frac{C_{e}}{q_{e}}=\left(\frac{1}{Q_{max}^{0}}\right)C_{e}+\frac{1}{Q_{max}^{0}K_{L}}$$

(5) $$\mathrm{Freundlich}\;\mathrm{isotherm}:\;logq_{e}=nlogC_{e}+logK_{F}$$

where *C_e_* (mg/L) is the fluoride concentration in equilibrium, *q_e_* (mg/g) is the amount of fluoride adsorbed at equilibrium, and *K_L_* (L/mg) is the Langmuir constant related to the affinity between an adsorbent and adsorbate, while *b*_1_ and *b*_2_ represent the two-site Langmuir constants; $Q_{max}^{0}$ (mg/g) is the maximum saturated monolayer adsorption capacity of an adsorbent, whereas *q*_1_ and *q*_2_ are the maximum uptake at high and low energy sites for the two-sites Langmuir constants. *K_F_* (mg/g)/(mg/L)^n^ is the Freundlich constant, and n (dimensionless) is the Freundlich intensity parameter.

The effect of pH on fluoride adsorption was studied by adjusting to a specific pH value in a range of 1–11 using 0.1 M HCl (≥36.5–38% Meyer, Química Suastes, S.A. de C.V., Mexico City, Mexico) or 0.1 M NaOH (CTR Scientific, Monterrey, Nuevo León, Mexico) with an initial fluoride concentration of 10 mg/L. The suspensions were shaken at 40 rpm for 180 min. The adsorbed fluoride ion concentration was determined as above described.

The effect of interfering anions (phosphate, nitrate, chloride, carbonate, bicarbonate, and sulphate) on fluoride adsorption was performed in batch tests with a fixed adsorbent load of 1.0 g/L and a fluoride concentration of 10.0 mg/L. The final concentrations of coexisting anions were 0.1, 1.0, and 10 mM, respectively, after using sodium salts (NaCl: ≥99%, Jalmek Científica, San Nicolás de los Garza, Mexico; NaNO_3_: ≥99%, Sigma-Aldrich Chemical Co., St. Louis, MO, USA; Na_2_SO_4_: ≥99%, Karal, León, Guanajuato, Mexico; Na_2_CO_3_: ≥99%, Karal, León, Guanajuato, Mexico; Na_3_PO_4_·12H_2_O: ≥98%, Karal, León, Guanajuato, Mexico; NaHCO_3_: ≥99%, Karal, León, Guanajuato, Mexico). The mixed suspensions were stirred at 40 rpm for 180 min. The adsorbed fluoride ion concentration was determined as described above.

## 3. Results

### 3.1. Characterization of Adsorbents

**Figure 1:**
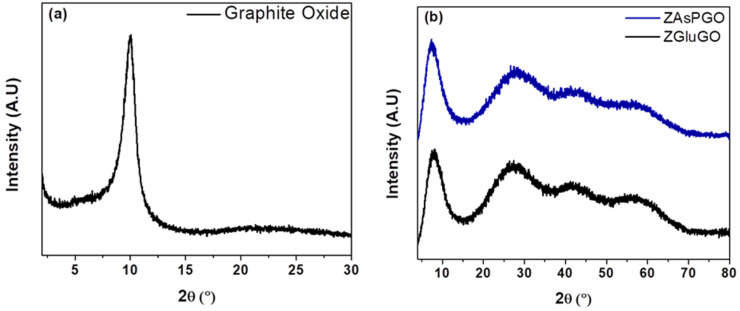
X-ray diffraction patterns for (**a**) graphite oxide, (**b**) ZGluGO and ZAspGO compounds.

ZAAGO compounds were characterized by different methods to obtain information about their morphological and chemical characteristics. Figure 1a shows the X-ray diffraction pattern of original graphite oxide. The characteristic GO peak can be seen at 10.1°. This indicates that the graphite oxide has a good degree of oxidation [40]. Figure 1b presents the diffractograms obtained for ZAsPGO and ZGluGO samples. The peaks at 7.4 and 8.1 degrees are related to the presence of graphite oxide in the samples. In addition, between 25° and 70°, there are three broad peaks. These patterns are in good agreement with previously reported amorphous zirconium oxide [43].

**Figure 2:**
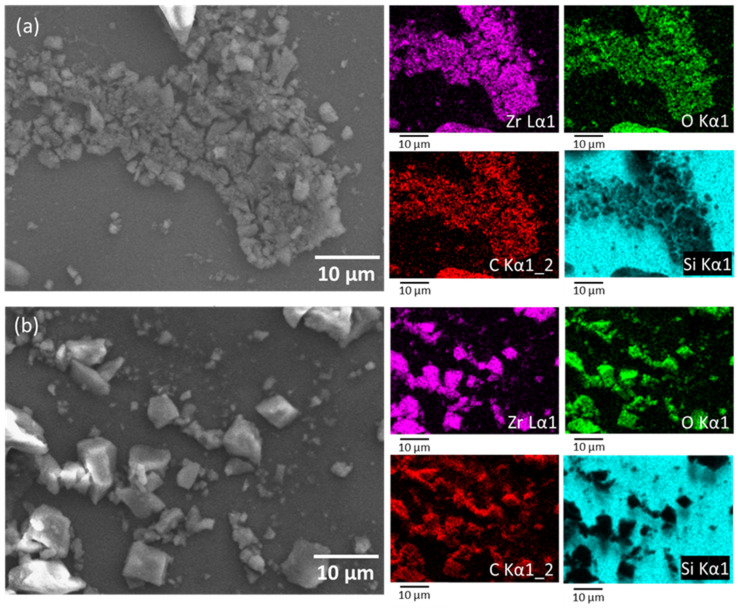
SEM-EDS elemental mapping images of (**a**) ZGluGO and (**b**) ZAspGO composites.

Scanning electron microscopy and energy-dispersive spectroscopy were employed to determine the surface morphology and the elemental distribution on the surface of ZAAGO compounds (Figure 2 and Figures S1–S4). According to SEM images, particles with broad size distribution were present in both samples without a specific morphology. The presence of the main elements, zirconium, oxygen, and carbon, was detectable in the samples, as shown with different colors in the mapping images (right side of Figure 2). The presence of silicon is due to the substrate used for SEM-EDS measurements. It is clearly seen that the elemental distribution of Zr in the carbon surface is homogenic. EDS spectra (Figures S3 and S4) showed similar Zr, O, and C content for both ZAAGO composites.

**Figure 3:**
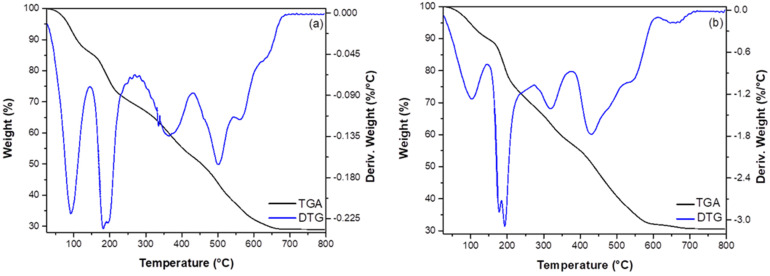
Thermogravimetric analysis of (**a**) ZGluGO and (**b**) ZAspGO samples.

ZGluGO and ZAspGO samples were characterized by TGA (see Figure 3). The combined graphs of thermogravimetry (TG) and its first derivative (DTG) show the stages of the thermal degradation of the adsorbent materials. The weight loss percentage analysis (TG graphs) shows four similar degradation steps for both compounds with weight loss of 9.4, 18.1, 17.2, and 26.2% for ZGluGO (Figure 3a, DTG graph: 92.6, 191.2, 364.8, and 502.6–563.6 °C, respectively) and 8.3, 15.5, 18.7, and 26.5% for ZAspGO (Figure 3b, DTG graph: 103.9, 185.1, 320.3, and 431.4–542.5 °C). The first degradation step presents the lowest percentage of weight loss (9.4 and 8.3%), which can be attributed to the evaporation of water on the surface of the material [44]. The second degradation step represents the loss of water of crystallization and the initial decomposition of organic components. In the third and fourth drop in the range of 250–600 °C, the thermal degradation corresponds to the amino acids [45] and to graphite oxide [46]. Around 600 °C, the residual mass varies within a range of 29–32%, assuming the formation of ZrO_2_. Therefore, we can argue that both adsorbent materials have similar behavior in terms of thermal stability.

**Figure 4:**
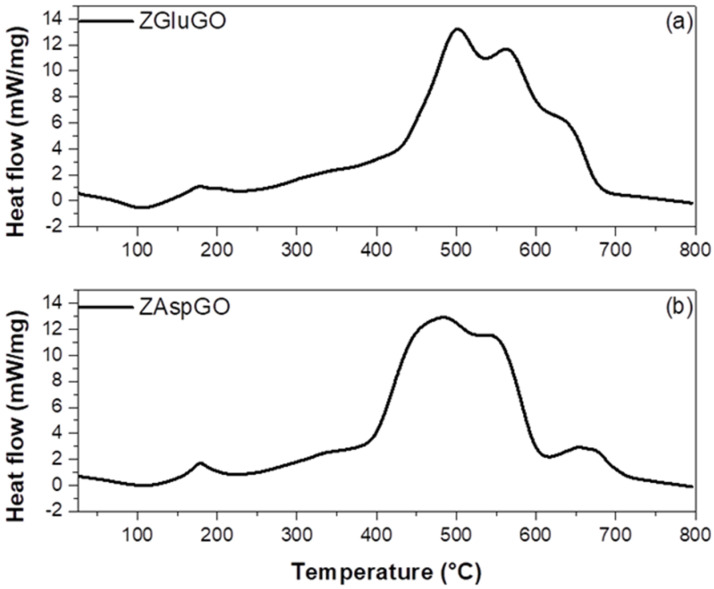
The differential scanning calorimetry plots of (**a**) ZgluGO and (**b**) ZaspGO samples.

DSC (differential scanning calorimetry) curves (Figure 4) for the adsorbent materials suggest the decomposition of ZGluGO and ZaspGO through an endothermic weight loss process that maximizes at 109.1 °C and 107.8 °C, respectively. This endothermic process is related to the dehydration of the sample, as well as the partial decomposition of the organic part. Subsequently, exothermic processes occur at 177.5, 331.7, 502.6, 562.6, and 633.6 °C for ZgluGO and at 178.8, 341.4, 483.4, 544.9, and 662.4 °C for ZaspGO, which may be related to the pyrolysis of the material, as well as dehydroxylation and crystallization of zirconium during heating [47].

**Figure 5:**
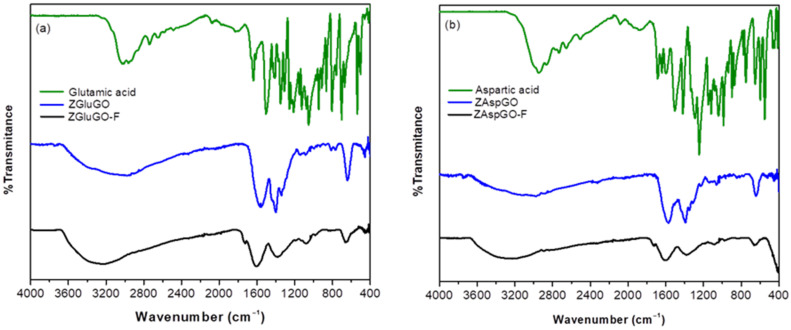
FTIR spectra of (**a**) glutamic acid, ZGluGO, and fluoride-treated ZGluGO (ZGluGO-F). (**b**) aspartic acid, ZAspGO, and fluoride-treated ZAspGO (ZAspGO-F).

The FTIR spectra were measured for the amino acids aspartic acid and glutamic acid, and the adsorbent materials ZgluGO and ZAspGO, before and after adsorbing the fluoride ion (Figure 5). The spectra obtained for the amino acids concur with the signals reported in the literature [48,49].

The characteristic band due to O-H stretching was located at 3140–3148 cm^−1^ for the two amino acids. On the other hand, the peaks of very low and medium intensity that appear at 3141 cm^−1^ and 3011 cm^−1^ were assigned to the asymmetric and symmetric stretching of N-H in aspartic acid, while in glutamic acid, they were present at 3139 cm^−1^ and 3020 cm^−1^, respectively. The medium-intensity bands located at 2950 cm^−1^ and 2863 cm^−1^ establish the presence of asymmetric stretches of C-H in the CH_2_ group of aspartic acid, and the symmetric-type CH_2_ stretches were observed at 2962 cm^−1^ and 2935 cm^−1^ for glutamic acid. For both compounds, the strong intensity peaks located at 1641 cm^−1^ and 1638 cm^−1^ were assigned to the asymmetric stretching of C=O, while the asymmetric stretching vibrations for this same type of bond were present between 1503 cm^−1^ and 1407 cm^−1^. Finally, for aspartic acid, the band located at 1689 cm^−1^ is assigned to the C=O-stretching and the NHN-bending frequencies [50,51].

After the coordination of GO and aspartic acid or glutamic acid to the zirconium atoms by carboxylate groups, it was observed that the characteristic signals of the amino acids (OH and NH_2_) overlapped, creating a wider signal with lower intensity in a range of 3500–3000 cm^−1^ due to the presence of water of hydration and crystallization and the formation of hydrogen bonds between the hydroxyl and amino groups. On the other hand, in the region where the signals corresponding to asymmetric vibration (ν_as_) and symmetric vibration (ν_s_) of the C=O groups were present, a slight displacement at a lower frequency is evident, which can be attributed to coordination of oxygen from the carboxyl group for the formation of chelates [52]. There are also two overlapping signals which can be attributed to the signals provided by the groups present at the surface of the graphite oxide and the amino acid. Likewise, a signal of about 649–621 cm^−1^ was identified, which is characteristic of the Zr-O interaction. When recording FTIR spectra of the compounds after adsorption, the same characteristic signals were observed as for the non-adsorbed compounds, except for the symmetric and asymmetric signals of C=O and C-O. The overlapping signals present in the bulk, pure compound disappeared and became simple, well-resolved signals, and there was a decrease in the signal assigned to the Zr-O vibrational mode, which may be attributed to the ligand exchange between the hydroxyl ion and the fluoride ion [30,31,53].

The “∆ criterion”, which is based on the difference between the values of the ν_as_ (COO) and ν_s_ (COO) vibration modes from the carboxylate group, was used to determine the coordination mode of this functional group [54,55]. If the difference between the asymmetric and symmetric vibrational modes of the carboxyl-type ligands in the synthesized compounds is greater than that of their ionic salts, there may be two types of interactions: if the value of ∆ is very large (>200 cm^−1^), this corresponds to a monodentate-type bond, whereas if the value is close to that of the ionic bond, it will be of the bridge type [56]. The ∆ν for the ZGluGO and ZAspGO compounds were 130, 231 cm^−1^ and 158, and 260 cm^−1^, respectively. The ∆ν data comparison of both compounds with the value of the purely ionic amino acid salt, sodium glutamate (∆ν_i_ = 78 cm^−1^), and sodium aspartate (∆ν_i_ = 108 cm^−1^) suggests the presence of monodentate and bridge-type coordinated COO groups between the metal centers of Zr (IV) and the aforementioned ligands, as well as possibly similar modes of coordination to GO. Table 1 shows the type of bonds proposed, as well as the ∆ values for the ligands in their ionic form and the synthesized compounds.

**Figure 6:**
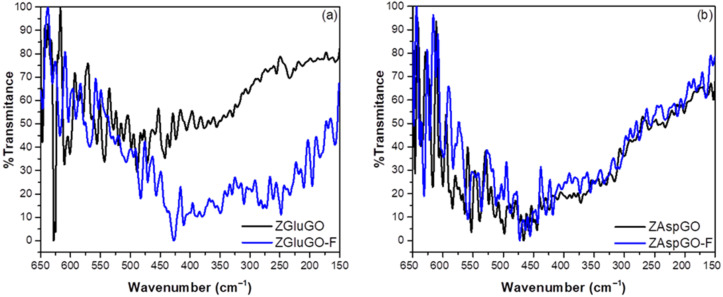
Far-infrared spectra of the compounds before and after fluoride ion adsorption: (**a**) ZGluGO and fluoride-treated ZGluGO (ZGluGO-F). (**b**) ZAspGO and fluoride-treated ZAspGO (ZAspGO-F).

Although the FTIR results corroborate the participation of surface hydroxyl groups and carboxyl groups in fluoride adsorption, there is no evidence for the role of metal nuclei of Zr(IV) in the adsorption of these ions, as these peaks are not apparent in the mid-range of infrared. Therefore, the far-infrared spectra of ZGluGO and ZAspGO were obtained before and after adsorption, exhibiting a series of peaks at a range of 650–150 cm^−1^, as shown in Figure 6.

The most intense signals that appeared in both compounds before adsorption were at 646 and 628–617 cm^−1^, attributed to Zr-OH vibrations. Likewise, low-intensity signals were identified for the translation of Zr-O n(O…O) located at 152–158, 162–168, 201–212, 216–233, 255–258, 269–283, and 298–302 cm^−1^ [57]. After adsorption, the intense bands present at wavelengths of 600 to 650 cm^−1^ related to the Zr-OH signal decreased considerably in intensity, most evidently for ZGluGO, as well as shifts in wave numbers for both compounds. The new signals resulting from these displacements are located at 638, 630, 621, 617, 611, and 603 cm^−1^, possibly related to a combination of the Zr–F and Zr–OH stretches. Asymmetric flexural vibrations ν_as_ (Zr–F) were attributed to a series of peaks located at 488–418 cm^−1^ and vibrations of type n(Zr–F) at 390–402 cm^−1^. Finally, a series of peaks located at a range of 246–356 cm^−1^ were assigned to the symmetric, asymmetric, and bending vibrations of the F–Zr–F type [58]. Based on these results, the Zr–F interaction after adsorption of fluoride ions can be confirmed.

### 3.2. Adsorption Studies

#### 3.2.1. Effect of pH

**Figure 7:**
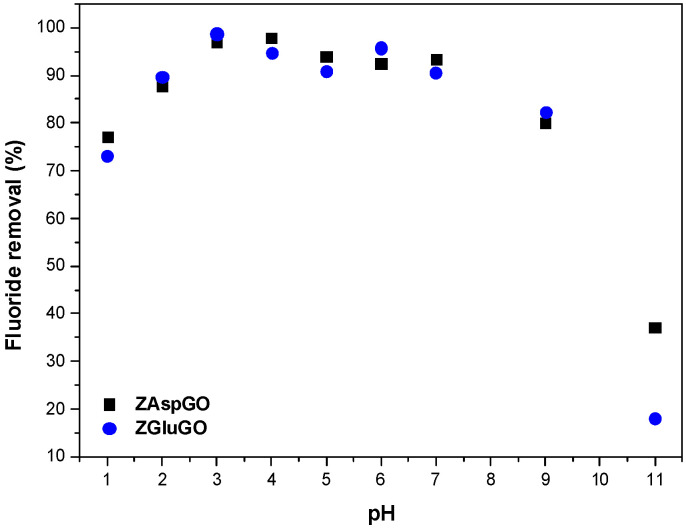
Effect of pH on the adsorption of fluoride ion by the ZAAGOs at 10 mg/L initial concentration.

Fluoride ion removal capacities were determined in a pH range of 1–11 for both ZAAGO adsorbents, maintaining an initial fluoride ion concentration of 10 mg/L. According to Figure 7, the highest removal capacities (>90%) were found to range between pH = 3 and 7, but they started to decrease above pH = 7, with a drastic decline above pH 10. The adsorbed amount was also remarkably lower (ca. 75%) at pH = 1. This pH-dependence strongly suggests that at least a part of the adsorption process is related to anion exchange. It is realistic to assume that the surface of the ZAAGO particles develops a positive charge by the protonation of pH-sensitive amino groups of the amino acid molecules exposed to the aqueous dispersion medium. The availability of the amino groups is most likely because the dicarboxylate-type amino acids can preferably coordinate to the zirconium ions by the aforementioned monodentate and bridge-type modes, leaving the amino groups available for interfacial protolytic reactions.

Since the protonation constants of amino groups in glutamic and aspartic acids are 9.7 and 9.8 [59], respectively, it is expected that in alkaline solutions with pH > 9 the positive surface charge density begins to decrease, and this must also result in a concomitant decrease in the exchangeable amounts of fluoride ions. Likewise, at low pH, the positive surface charge also decreases gradually owing to the more extensive charge screening of the ions present in the solution upon the addition of acid (chloride and H_3_O^+^).

#### 3.2.2. Adsorption Isotherms

Quantitative characterization of the immobilization of fluoride ions is possible by constructing solid/liquid (S/L) adsorption isotherms. These functions, in a strict sense, relate to the surface excess concentration of an adsorbate as a function of its bulk concentration, under equilibrium conditions and constant temperature [60]. In dilute solutions, the surface excess concentration can be approximated by the total number of moles of adsorbate per unit surface area of the solid. Because the surface area exposed to the S/L interaction is usually not determined or only approximated by nitrogen adsorption, most experimental studies on S/L adsorption comfortably use the quantity termed “adsorbed amount” (*q_e_*). This quantity signifies the mass of adsorbate per unit mass of the adsorbent, typically in mg/g units.

**Figure 8:**
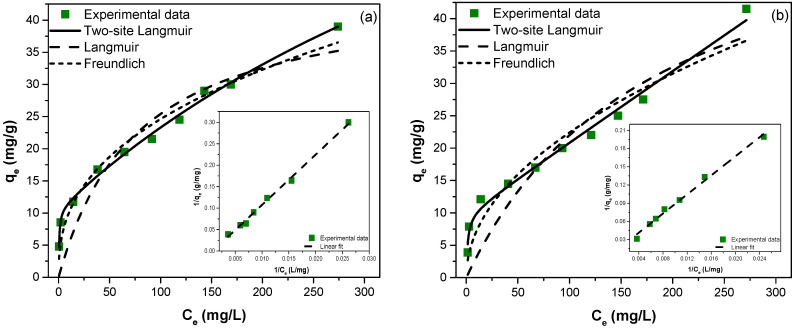
Adsorption isotherms, nonlinear fitting models of two-site Langmuir, Langmuir, and Freundlich isotherms, and linear fitting model of two-site Langmuir isotherm (insert) for fluoride removal (**a**) ZGluGO and (**b**) ZAspGO at 25 °C.

The left panel of Figure 8 shows the adsorption isotherm of fluoride ions on ZGluGO. Most noteworthy, the adsorbed amount of F^−^ shows an abrupt increase at undetectably low solution concentrations up to *q_e_* ~ 10 mg/g. Then, it is increased in a nearly linear fashion until quite high equilibrium concentrations, without any indication of reaching a saturation adsorption, even at *c_e_* >250 mg/L. Regarding the adsorption on ZAspGO (right panel of Figure 8), it is also evident that the extent of fluoride ion binding proceeds in a very similar fashion, because both the shape of the isotherm and the respective *q_e_* values are identical to those of the ZGluGO composite.

While the experimental isotherms provide practically relevant information on the extent of adsorption and removal capabilities of the zirconium-based composites towards fluoride anions in the ppm concentration range, the saturation (maximum) adsorption cannot be deduced from them. Therefore, we performed several curve-fitting protocols to assess the maximum adsorbed amount ($Q_{max}^{0}$, *q*_1_, *q*_2_) as well as an “interaction parameter”, which is representative of the binding strength between the surface sites and the F^−^ ions. Three isotherm models were tested: (i) Langmuir model (Equation (1)), (ii) Freundlich model (Equation (2)), and (iii) two-site Langmuir model (Equation (3)).

The maximum monolayer adsorption capacities obtained from the linear and nonlinear models of the Langmuir isotherm for ZGluGO and ZAspGO were 38.76, 45.28 mg/g and 39.37, 62.46 mg/g, respectively. In addition, it was found that the equilibrium parameter (*R_L_*) values vary within a range of 0.116–0.976 for the linear and nonlinear fit, at an initial F^−^ concentration of 5–300 mg/L. These values are in the range of 0–1, which indicates a favorable adsorption process. On the other hand, the values of n in the Freundlich equation were calculated as 0.299, 0.392 and 0.363, and 0.491 for the linear and nonlinear adjustments of the adsorbent materials ZGluGO and ZAspGO. Since these values are between zero and one, this also indicates a favorable adsorption of fluoride on the surface of these materials. However, although there was a good correlation between the constant *R_L_* and the parameter n of the Langmuir and Freundlich models, the study of the fluoride adsorption isotherms showed that the experimental data have a better fit in the linear form and nonlinear for the two-site Langmuir model, obtaining values of *R*^2^ close to 1 (0.9830–0.9959), as well as lower values of χ^2^ (0.705–1.976) (Figure 8 and Table 2. Linear fitting of Langmuir and Freundlich isotherms present in Figures S5 and S6). The maximum adsorption capacity obtained for this model in the adsorbent materials ZGluGO and ZAspGO was 105.3 and 101.0 mg/g in the linear form, respectively, and around 108 mg/g in the nonlinear form.

#### 3.2.3. Interfering Ions

**Figure 9:**
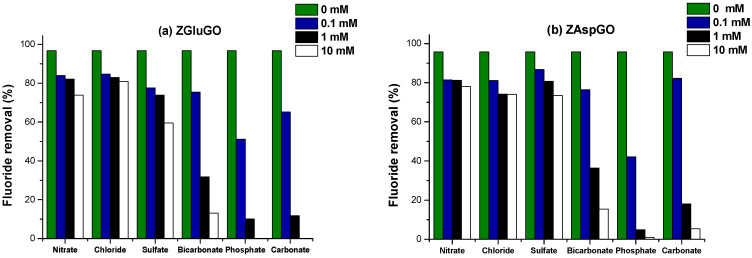
Effect of interfering anions with different concentrations (0.1, 1, and 10 mM) on the fluoride ion-removal efficiencies (F^−^ initial concentration was constant, 10 mg/L) (**a**) ZGluGO and (**b**) ZAspGO at 25 °C.

Natural waters always contain a great diversity of minerals, such as chloride, sulphate, nitrate, bicarbonate, and phosphate, against which the fluoride ion can compete. Likewise, these may reduce the capacity of the adsorbent to remove the fluoride ion. Different concentrations of the interfering ion were used (0 mM, 0.1 mM, 1 mM, and 10 mM). The results are presented in Figure 9, where it is apparent that adsorption capacity is not significantly influenced by nitrate, chloride, or sulphate ions. This concurs with studies reported in the literature [61,62]. Although the bicarbonate and carbonate ions presented a decrease of more than 50% at a concentration greater than 1 mM, this is because these ions can interact with zirconium and form a stable pentacyclic complex with the Zr^4+^ which occupies active sites, competing for the adsorption of the fluoride ion, thus negatively affecting its removal [31,63]. However, the phosphate ion showed inhibition of the fluoride ion because of the presence of OH^-^ ions formed during the hydrolysis of Na_3_PO_4_; the ratio of hydroxyl ions increases the pH of the aqueous medium, converting it into a basic medium. Likewise, these generated ions represent competition for the active sites of the compound [31,63].

Different zirconium-containing fluoride ion adsorbents were compared, as shown in Table 3. ZGluGO and ZAspGO compounds had a significantly higher adsorption capacities than other composites. There was no remarkable difference in the functional pH range. The fluoride ion adsorption was notably affected in the presence of bicarbonate ion in almost every case.

## 4. Conclusions

Zr-amino acid-graphite oxide composites were successfully synthesized by the chemical precipitation method using a suspension of graphite oxide, negatively charged side-chain amino acids (aspartic acid and glutamic acid), and ZrOCl_2_, and tested for fluoride ion removal for the first time. Both the carbon counterpart of the composite and the amino acid molecules can form stable complexes with the zirconium centers and constitute a structurally stable solid framework for the removal of fluoride ions in aqueous solution in a wide pH range. The adsorption process presented a better fit for the two-site Langmuir isotherm in its linear and nonlinear forms compared to the Langmuir and Freundlich models. It is proposed that the mechanisms of fluoride removal using ZGluGO and ZAspGO complexes involve both ion-exchange and ligand-exchange processes, which is supported by isotherm models and spectroscopic evidence. A maximum adsorption capacity of between 101 and 105 mg/g in a contact time of 180 min at neutral pH was found, showing that both compounds could remove fluoride ions efficiently and in a reasonable time. These extremely high adsorbed amounts can be achieved only from very concentrated fluoride ion-containing solutions, representing only the most heavily contaminated volcanic or mountainous groundwaters such as those found in India (up to 400 mg/L F^−^) [65]. However, the usual permissible limits of F^−^ in drinking waters (1.5 ppm) can be safely and efficiently achieved for most of the globally occurring over-fluoridated groundwater sources by the developed ZAAGO adsorbents because they can take up fluoride anions in a virtually irreversible way up to a 10 mg/g capacity. The effects of different interfering ions commonly found in surface and groundwater were studied, of which nitrate, chloride, and sulphate ions had negligible impact on the performance of the process. Finally, we need to mention that the costs associated with the mass production of the present adsorbents are high because of the amino acid counterparts, and they cannot currently compete with other cheaper Zr-based adsorbents such as Zr-oxyhydroxides. However, we highlight that whenever a compound gains a significant interest in application, its mass production becomes fundamentally more cost effective upon the development of appropriate synthesis strategies. Glutamic acid is already produced on the largest scale of any amino acid, with an estimated annual production of about 1.5 million tons in 2006 [66]. Therefore, it can be concluded that the obtained adsorbent materials can be used for the development of technology applied to the removal of contaminants in water.

## Figures and Tables

**Table 1:** Relationships between the asymmetric and symmetric vibrational modes of ZGluGO and ZAspGO.

Compound	∆ (ν_as_ − ν_s_)	Bond Type
Sodium glutamate	78	Ionic
ZGluGO	231	Monodentate
	130	Bridge
Sodium aspartate	108	Ionic
ZAspGO	260	Monodentate
	158	Bridge

**Table 2:** Calculated parameters for fluoride-adsorption isotherm models of ZGluGO and ZAspGO.

Material	Isotherm Model	Parameters					Statistics	
							*R* ^2^	χ^2^
	Langmuir	$Q_{max}^{0}$	*K_L_*			*R_L_*		
		(mg/g)	(L/mg)					
ZGluGO	Nonlinear	45.28	0.013			0.204–0.939	0.8492	16.81
	Linear	38.76	0.025			0.116–0.88	0.9070	–
ZAspGO	Nonlinear	62.46	0.005			0.400–0.976	0.8154	21.51
	Linear	39.37	0.018			0.153–0.915	0.8243	–
	Freundlich	*K_F_*	n					
		(mg/g)						
ZGluGO	Nonlinear	4.044	0.392			–	0.9653	3.865
	Linear	6.112	0.299			–	0.9728	-
ZAspGO	Nonlinear	2.333	0.491			–	0.9205	9.260
	Linear	4.218	0.363			–	0.9448	–
	Two-site Langmuir	*q*_1_	*b*_1_	*q*_2_	*b*_2_			
		(mg/g)	(L/mg)	(mg/g)	(L/mg)			
ZGluGO	Nonlinear	97.32	0.0015	10.64	1.8547	–	0.9937	0.705
ZAspGO		–	–	9.924	0.7663	–	0.9830	1.976
		*q*_1_ + *q*_2_		*b*_1_ + *b*_2_				
ZGluGO	Linear	105.3		0.0008		–	0.9959	–
ZAspGO		101.0		0.0013		–	0.9927	–

**Table 3:** Comparison of different zirconium-containing fluoride ion adsorbents (AC: adsorption capacity; pH: functional pH range; CT: contact time).

Adsorbent	AC (mg/g)	pH	CT (min)	Interfering Ions	Reference
Oxalic acid-mediated polyacrylamide-zirconium complex	9.6	3–7	20	HCO_3_^−^	[30]
Oxalic acid-graphene oxide-zirconium complex	9.7	3–7	18	HCO_3_^−^	[31]
Zirconium-oxalic acid-activated carbon	7.4	7	50	Mixture of Cl^−^, SO_4_^2−^, PO_4_^3−^, NO_3_^−^ and HCO_3_^−^	[39]
Zirconium-impregnated magnetic chitosan-graphene oxide	8.84	4–8	180	HCO_3_^-^	[64]
Zirconium-modified activated carbon fibers	28.5	3.2–5.6	360	-	[29]
ZGluGO	105.3	3–7	180	HCO_3_^−^, CO_3_^2−^ and PO_4_^3−^	Present work
ZAspGO	101.0	3–7	180	HCO_3_^−^, CO_3_^2−^ and PO_4_^3−^	Present work

## Data Availability

The data presented in this study are available on request from the corresponding author.

## Supplementary Materials

The following supporting information can be downloaded at: https://www.mdpi.com/article/10.3390/ijerph19063640/s1, Figure S1. SEM images of ZGluGO composite, Figure S2. SEM images of ZAspGO composite, Figure S3. SEM image and EDS spectrum of ZGluGO composite, Figure S4. SEM image and EDS spectrum of ZAspGO composite, Figure S5. Linear fitting model of Langmuir isotherm for fluoride removal (a) ZGluGO and (b) ZAspGO, Figure S6. Linear fitting model of Freundlich isotherm for fluoride removal (a) ZGluGO and (b) ZAspGO.
