# Supplementary material for: Amino Acid Complexes of Zirconium in a Carbon Composite for the Efficient Removal of Fluoride Ions from Water
Source: Int J Environ Res Public Health. 2022 Mar 18;19(6):3640. doi: 10.3390/ijerph19063640 (PMC8952250; doi:10.3390/ijerph19063640)
Supplement: Supplementary file 1 [file ijerph-19-03640-s001.zip › ijerph-1598124-supplementary.pdf]

Supplementary Materials

Amino Acid Complexes of Zirconium in a Carbon Composite for the Efficient Removal of Fluoride Ions from Water

Efrén González-Aguiñaga [1], José Antonio Pérez-Tavares [1], Rita Patakfalvi [1,*], Tamás Szabó [2,*], Erzsébet Illés [3], Héctor Pérez Ladrón de Guevara [1], Pablo Eduardo Cardoso-Avila [4], Jesús Castañeda-Contreras [1] and Quetzalcoatl Enrique Saavedra Arroyo

Centro Universitario de los Lagos, Universidad de Guadalajara, Lagos de Moreno 47460, Jalisco, Mexico; efren.gonzalez5990@alumnos.udg.mx (E.G.-A.); ptavaresjose@gmail.com (J.A.P.-T.); hperez@culagos.udg.mx (H.P.L.d.G.); jesus.castaneda@academicos.udg.mx (J.C.-C.)
[2]Department of Physical Chemistry and Materials Science, University of Szeged, Rerrich Béla tér 1, H-6720 Szeged, Hungary; sztamas@chem.u-szeged.hu (T.S.)
[3]Department of Food Engineering, University of Szeged, 6720 Szeged, Hungary; illese@chem.u-szeged.hu
[4]Centro de Investigaciones en Óptica, A.C, León 37150, Guanajuato, Mexico; pecardoso@cio.mx
[5]Instituto Tecnológico Superior de Irapuato, Irapuato 36821, Guanajuato, Mexico; enrique.sa@irapuato.tecnm.mx
*Correspondence: rita.patakfalvi@academicos.udg.mx (R.P); sztamas@chem.u-szeged.hu (T.S.)

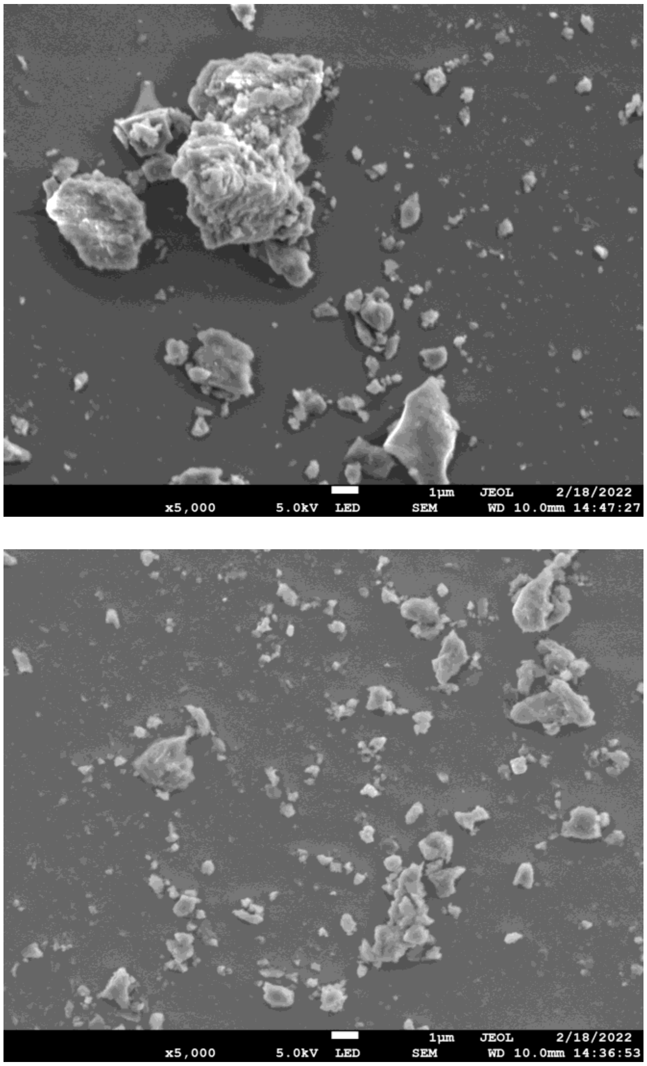

Figure S1. SEM images of ZGluGO composite

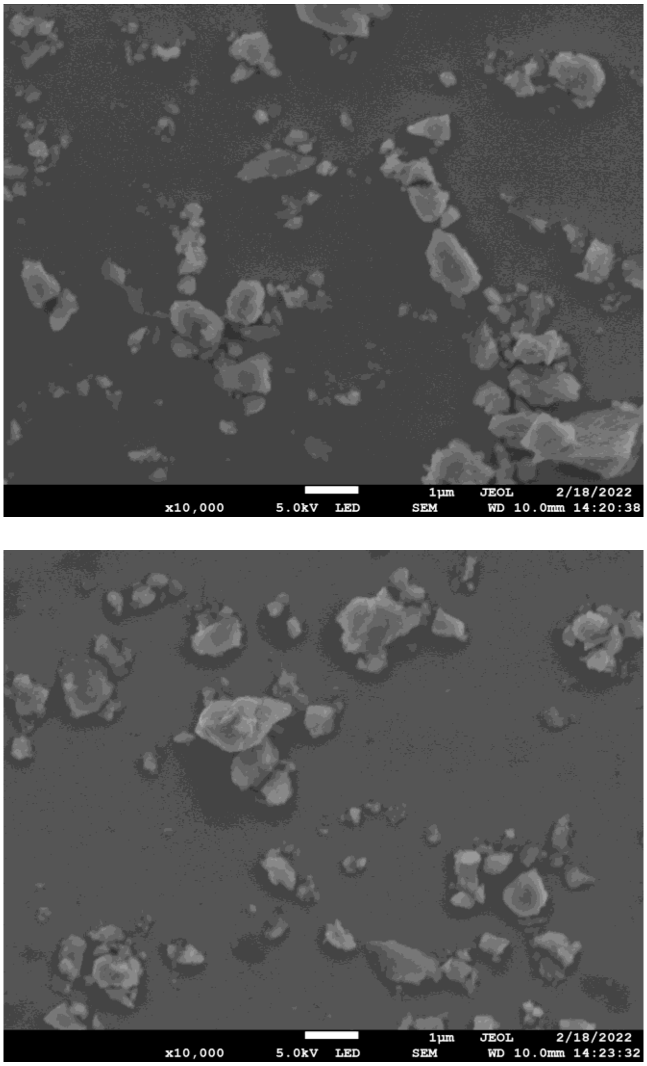

Figure S2. SEM images of ZAspGO composite

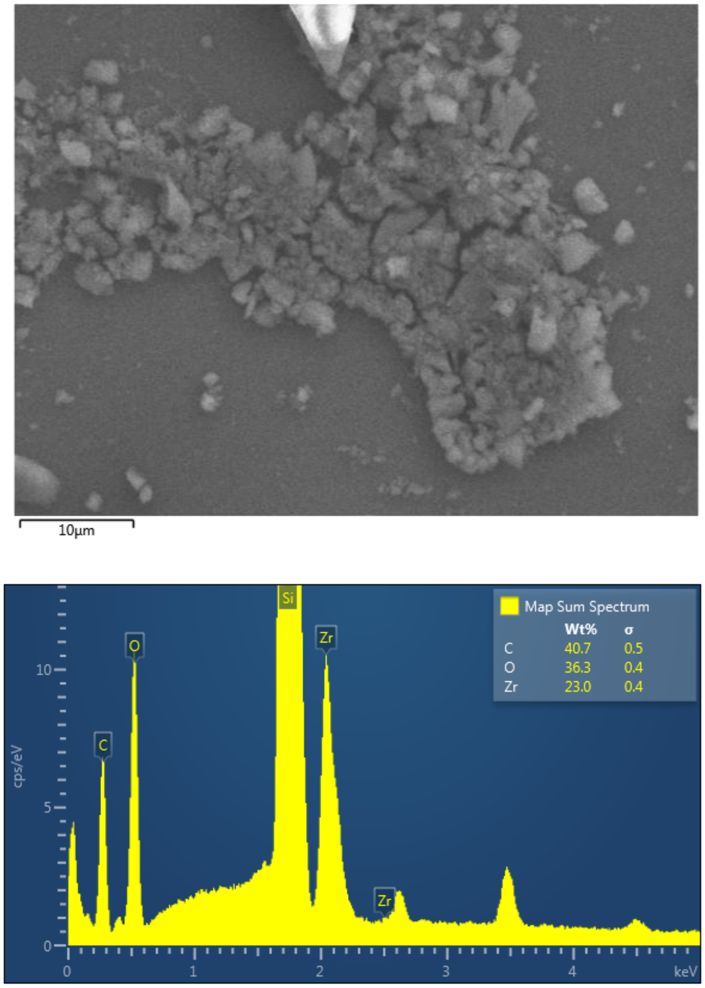

Figure S3. SEM image and EDS spectrum of ZGluGO composite

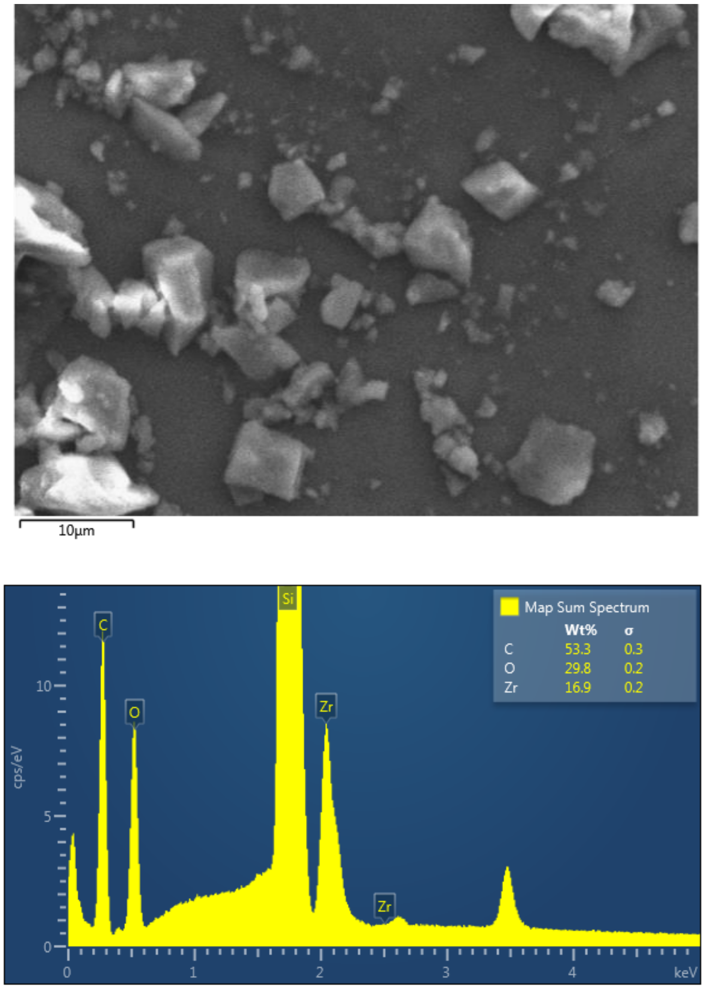

Figure S4. SEM image and EDS spectrum of ZAspGO composite

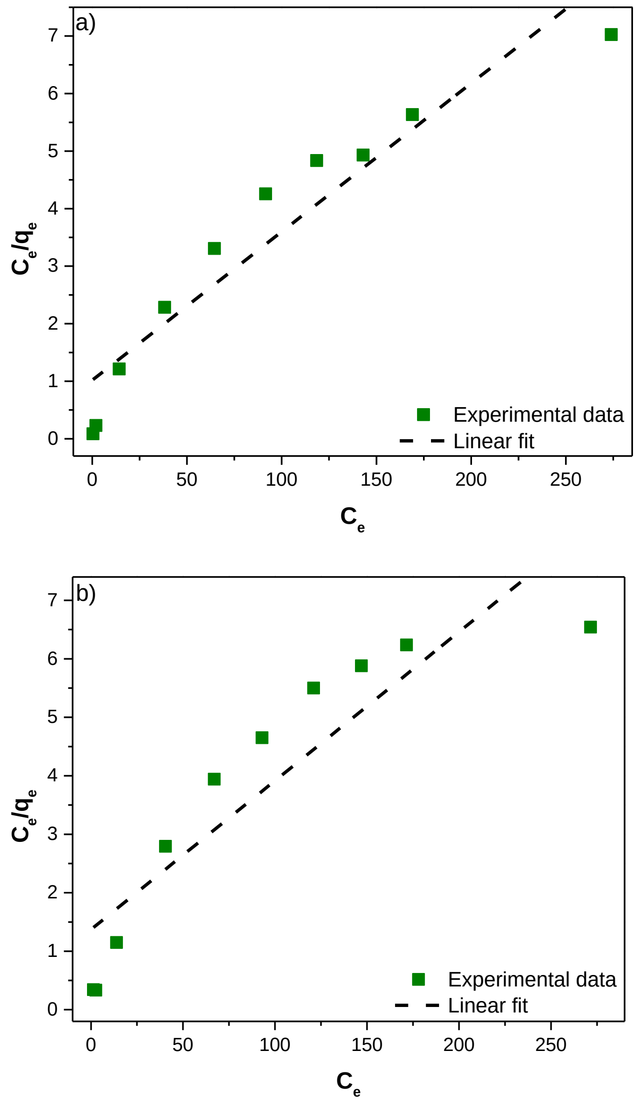

Figure S5. Linear fitting model of Langmuir isotherm for fluoride removal (**a**) ZGluGO and (**b**) ZAspGO

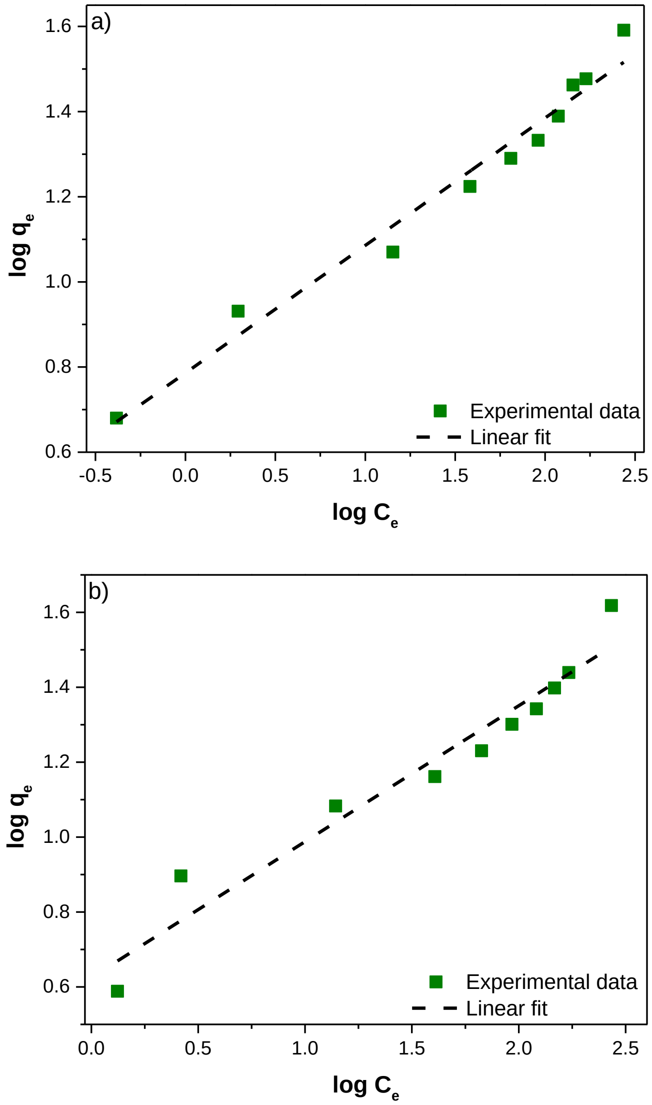

Figure S6. Linear fitting model of Freundlich isotherm for fluoride removal (**a**) ZGluGO and (**b**) ZAspGO
